# Contributions of Pavlovian incentive motivation to cue-potentiated feeding

**DOI:** 10.1038/s41598-018-21046-0

**Published:** 2018-02-09

**Authors:** Andrew T. Marshall, Briac Halbout, Angela T. Liu, Sean B. Ostlund

**Affiliations:** Department of Anesthesiology and Perioperative Care, Center for Addiction Neuroscience, University of California, Irvine, Irvine, CA USA

## Abstract

Cues signaling the availability of palatable food acquire the ability to potentiate food seeking and consumption. The current study employed a combination of behavioral, pharmacological, and analytical techniques to probe the role of Pavlovian incentive motivation in cue-potentiated feeding. We show that a cue paired with sucrose solution (CS+) can transfer its control over feeding to stimulate sucrose consumption at a new receptacle, and that this effect depends on activation of D1 dopamine receptors, which is known to modulate other forms of cue-motivated behavior but not taste palatability. Microstructural analyses of sucrose-licking behavior revealed that the CS+ tended to increase the frequency with which rats engaged in active bouts of licking behavior without having a reliable effect on the duration of those licking bouts, a measure that was instead associated with sucrose palatability. Furthermore, we found that individual differences in CS+ elicited increases in bout frequency were associated with total sucrose intake at test, supporting the view that this process was related to meaningful dysregulation of eating behavior. The current study, therefore, (1) demonstrates that a dopamine-dependent Pavlovian incentive motivational process can mediate cue-potentiated feeding, and (2) lays out an experimental and analytical approach for parsing this aspect of behavior.

## Introduction

Environmental cues that signal the availability of palatable foods can trigger powerful food cravings^1–3^ and promote eating in the absence of hunger, an effect observed in rodents^4,5^ and humans^6–9^. This behavioral influence, which is believed to play an important role in overeating and obesity^10–13^, can be studied using the cue-potentiated feeding (CPF) task. In a typical CPF study, hungry animals undergo Pavlovian conditioning consisting of repeated pairings between a conditioned stimulus (CS+; e.g., an auditory tone) and a small quantity of a palatable food or fluid, such as sucrose solution, which they consume from a cup located in a fixed position in the experimental chamber. Next, they are given unrestricted access to their maintenance chow to ensure that they are fully sated prior to testing. Animals are then returned to the chamber and allowed to freely consume sucrose from the cup while the CS+ is intermittently presented in a noncontingent manner. Under such conditions, animals exhibit a pronounced elevation in food consumption during test sessions with the CS+ relative to sessions with an unpaired stimulus (CS−).

While such findings indicate that external cues can act independently of physiological hunger to promote feeding, the psychological processes underlying this effect are not firmly established. One possibility is that cues associated with palatable food consumption acquire reflexive or habitual control over feeding (i.e., stimulus-response based). If this is the primary mechanism mediating CPF, then the CS+ should stimulate consumption by eliciting the specific feeding behaviors that were established during Pavlovian conditioning. This *response learning* view is plausible when the source of food is fixed across training and testing, as in the example described above. Although this scenario applies to most demonstrations of CPF, there have also been reports that food-associated cues can trigger feeding at new locations^14–16^, indicating that they can control feeding indirectly. One possible explanation is that such cues potentiate feeding through the same Pavlovian incentive motivational process that allows them to elicit and invigorate instrumental food-seeking behaviors^17,18^. This *motivational view* predicts that the CS+ triggers an urge to search for food, which would also lead to feeding when food is readily available. Alternatively, given evidence that signals for palatable food can enhance hedonic evaluation of taste stimuli^19–21^, it is possible that cues potentiate feeding in part by making food more palatable. While this hedonic view is mechanistically distinct from the motivational view, these accounts are not mutually exclusive and may explain different aspects of CPF^10,22^.

One way to distinguish between the motivational and hedonic accounts of CPF is to determine how food-paired cues influence the microstructure of feeding. When rodents are allowed to freely consume sucrose solution or other palatable fluids, they engage in licking bouts of varying durations that are separated by periods of inactivity. Whereas the average duration of these licking bouts provides a reliable and selective measure of fluid palatability^23,24^, it is believed that the frequency of these bouts is controlled by motivational processes^25–28^. Thus, if the CS+ stimulates feeding by enhancing sucrose palatability, then that cue should increase the duration, but not necessarily the frequency, of licking bouts. In contrast, the motivational view predicts that the CS+ should trigger sucrose seeking and consumption even when animals are preoccupied with other activities, leading to more frequent, but not necessarily longer, bouts of licking.

The current study investigated the effects of CS+ delivery on sucrose licking microstructure using two CPF protocols, one in which sucrose was always available at the same location (Experiment 1), and one in which the source was changed across training and testing (Experiments 2 and 3), allowing us to evaluate the indirect influence of the CS+ . Our approach for assessing this response-independent (generalized) influence of food-paired cues on feeding was modeled after the Pavlovian-to-instrumental transfer (PIT) task, which is widely used to study the incentive motivational impact of reward-paired cues on reward-seeking behavior^18,29,30^. We also adopted Pavlovian conditioning and testing parameters commonly used in PIT studies to facilitate comparison with that literature. Given that dopamine D1 receptor activity is crucial for expression of PIT and other measures of cue-motivated behavior^31–33^ but is relatively unimportant for hedonic aspects of feeding behavior^25,28,34^, we also assessed the impact of D1 receptor blockade on cue-potentiated sucrose licking (Experiment 3) as a further probe of the role of motivation in this effect. Finally, we analyzed the microstructure of sucrose licking data from these experiments to test whether CPF was selectively associated with increases in either the frequency or duration of bouts of sucrose licking, as would be predicted by the motivational and hedonic views of CPF, respectively.

## Results

### Cue-potentiated feeding with a cue that signals the food source

In our first experiment, we applied a conventional response-congruent CPF design, in which the specific responses required to consume sucrose were the same across training and testing phases. Hungry rats were given 10 d of Pavlovian conditioning to establish the CS+ as a cue for sucrose availability at a food cup on one side of the chamber. By the last day of conditioning, cup entries (±between-subjects SEM) were significantly higher during the CS+ (23.72 ± 2.79 per minute) versus the inter-trial interval [18.27 ± 3.25 per minute; paired-samples *t*-test, *t*(15) = 3.13, *p* = 0.007]. Cup entries during the CS− (8.60 ± 1.91 per minute) did not significantly differ from the inter-trial interval [10.69 ± 2.00 per minute; paired-samples *t*-test, *t*(15) =−1.60, *p* = 0.130].

**Figure 1:**
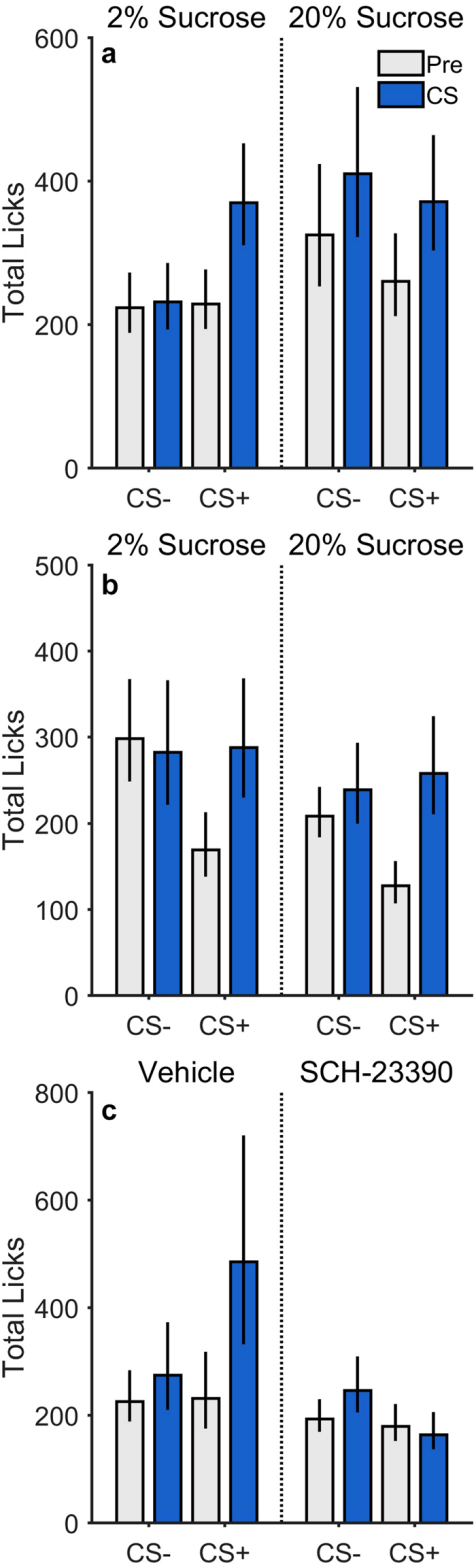
Total licking behavior. Results of Experiments 1–3 (**a–c**, respectively) assessing the impact of a sucrose-paired cue (CS+) and unpaired cue (CS−) on sucrose licking at (**a**) the same food cup used during Pavlovian conditioning, and (**b**,**c**) an apparatus (bottle) and location not used during Pavlovian conditioning. Data are displayed as a function of CS period (Pre, CS), CS type (+,−), and concentration (**a**,**b**) or drug (**c**). Error bars represent ±1 standard error of the estimated marginal means from the fitted generalized linear mixed-effects model.

Rats were then given two CPF tests in a food-sated state to characterize the effects of the CS+ on sucrose licking. In each test, rats had free access to 2% or 20% sucrose solution, allowing us to assess the influence of sucrose palatability on CPF. Figure 1a shows the total number of licks observed during CS trials as a function of CS period, CS type, and sucrose concentration. Data were analyzed using generalized linear mixed effects models (Supplementary Table S1). Importantly, there was a significant CS Period × CS Type interaction, *t*(116) = 12.70, *p* < 0.001. Further analysis (collapsing across concentration) revealed a significant increase for CS+ trials, *p* < 0.001, but not CS− trials, *p* = 0.118, indicating that the CS+ was more effective than the CS− in increasing sucrose licking, relative to pre-CS levels. Our analysis also found that this cue-selectivity was significantly influenced by sucrose concentration (3-way interaction, *p* < 0.001). Specifically, although the CS+ was highly effective in elevating sucrose licking in both 2% and 20% conditions, *p*s < 0.001, the CS− did not significantly affect lick rates in the 2% test, *p* = 0.309, but provoked a modest but significant increase in the 20% test, *p* = 0.039. Thus, although the food-paired cue was generally more effective in controlling feeding, the unpaired cue seemed to exert a similar influence when rats were allowed to consume a highly palatable sucrose solution at test.

### Transfer of cue-potentiated feeding to a new food source

Because sucrose was made available at the same source during training and testing in Experiment 1, it is unclear whether the observed CPF effect was dependent on the ability of the CS+ to (1) *motivate* rats to seek out and consume sucrose or (2) directly elicit a specific *conditioned reflex*, or *habit*. Experiment 2 focused more squarely on the former hypothesis by testing whether a CS+ associated with delivery of sucrose into a food cup could motivate sucrose licking from a spout on the opposite side of the chamber at test, comparable to behavioral phenomena observed in PIT.

Rats were trained with the same Pavlovian conditioning procedure used in Experiment 1, resulting in cue-specific anticipatory approach behavior by the last day of Pavlovian conditioning. Food cup approaches (± between-subjects SEM) were greater during the CS+ (18.71 ± 1.73 per minute) relative to the inter-trial interval [12.49 ± 0.98 per minute; paired-samples *t*-test, *t*(15) = 3.02, *p* = 0.009]. There were no significant differences between the CS− (9.41 ± 0.98 per minute) and the inter-trial interval [8.44 ± 0.88 per minute; paired-samples *t*-test, *t*(15) = 0.98, *p* = 0.341].

Given that the effects of the CS+ on sucrose licking in Experiment 1 were somewhat more apparent when rats were tested with 2% sucrose, our initial testing with sucrose available at a new source (spout, with food cup covered by an opaque panel – See *Methods*) focused on this condition. However, in this test, sucrose licking did not significantly differ between CS+ (328.1 ± 84.8 licks) and pre-CS+ periods [245.6 ± 45.9 licks; paired-samples *t*-test, *t*(15) = 1.07, *p* = 0.300]. To further discourage response competition and strengthen sucrose drinking from the spout, rats were given 5 additional sessions of training to lick from the spout for 20% sucrose under food deprivation in the absence of the CSs. Rats were then fully sated on home chow and administered two CPF tests with sucrose available at the metal spout. During tests, rats had continuous access to 2% or 20% sucrose solution in separate tests (within-subjects, order counterbalanced).

Figure 1b shows that during this round of testing, the CS+ was effective in promoting sucrose drinking at the new location, even though that cue was never directly associated with this behavior. Mixed-effects model analysis (Supplementary Table S2) found a significant CS Type × CS Period interaction, *t*(120) = 15.16, *p* < 0.001, indicating that the CS+ was more effective in elevating sucrose licking over baseline levels (CS vs. pre-CS period, *p* < 0.001) than the CS− (CS vs. pre-CS period, *p* = 0.097), as in Experiment 1. Sucrose concentration did not significantly influence the cue-selectivity of this effect (3-way interaction, *p* = 0.319). Importantly, while lick rates appeared to be elevated during the pre-CS− relative to the pre-CS+ periods, paired-samples *t*-tests indicated that this difference was not statistically significant in the 2% condition, *t*(15) = 1.66, *p* = 0.118, or in the 20% condition, *t*(15) = 1.56, *p* = 0.139. This is to be expected given the pseudo-random trial structure used during training and testing, which prevents systematic (inter-trial) carryover effects and precludes anticipation of future trial type (or timing). It is also worth noting that these same animals showed similar CS+ specific elevations in licking in Experiment 3 when their pre-CS− and pre-CS+ lick rates were more comparable (see Fig. 1c, vehicle).

### Dependence on D1-type dopamine receptors

The results of Experiment 2 indicate that the CS+ acquired the ability to potentiate sucrose consumption by triggering a feeding behavior that had never directly been associated with that cue, consistent with a PIT-like motivational influence. Given the importance of D1-type dopamine receptors in Pavlovian incentive motivation^31–33^, Experiment 3 examined whether blocking activity at these receptors would disrupt CPF expression. The same rats used in Experiment 2 were given a final pair of CPF tests (20% sucrose) after pretreatment with SCH-23390 (0.04 mg/kg), a selective D1 antagonist, or vehicle. Test results are shown in Fig. 1c (also Supplementary Table S3).

Analysis revealed a main effect of drug treatment, *t*(120) = −2.15, *p* = 0.034, in that sucrose licking was generally depressed by SCH-23390. Importantly, we found a significant Drug × CS Period × CS Type interaction, *t*(120) = −20.91, *p* < 0.001, indicating that SCH-23390 specifically disrupted the expression of CPF. Indeed, further analysis revealed that while the CS+ significantly increased sucrose licking over pre-CS+ levels in the vehicle test, *p* < 0.001, there was no effect of the CS+ in the SCH-23390 test, *p* = 0.982. Similar to the cue generalization observed in Experiment 1, the CS− elicited marginally significant increases in sucrose licking in both drug conditions, *p*s ≤ 0.049. Thus, D1-type dopamine receptor antagonism via SCH-23390 administration significantly impaired CS+ evoked feeding, consistent with an incentive motivational account of CPF.

### Microstructural analysis of the effects of sucrose-paired cues and sucrose concentration on feeding

The results of Experiments 2 and 3 suggest that the novel PIT-like protocol used here supports an incentive motivational form of CPF, as the cues were able to motivate feeding behavior at a location separate from the food source signaled by the cue. To further test this account, we examined if the excitatory effects of the CS+ on sucrose drinking were related to a specific change in the microstructural organization of licking behavior. As described above, whereas licking bout duration varies with fluid palatability^23,24^, the frequency with which rats engage in new bouts of licking is thought to reflect a separate motivational process^25–28^. We varied sucrose concentration to manipulate its palatability, as in previous reports^23,35^. Although high and low sucrose concentrations also differ in caloric content, extensive research has shown that the bout duration measure is a sensitive and selective measure of the influence of orosensory reward and is dissociable from post-consummatory calorie processing^35–38^. Thus, a CS+ that induces incentive motivation should increase bout frequency, while a CS+ that increases intake by making sucrose more palatable should promote longer bout durations.

**Figure 2:**
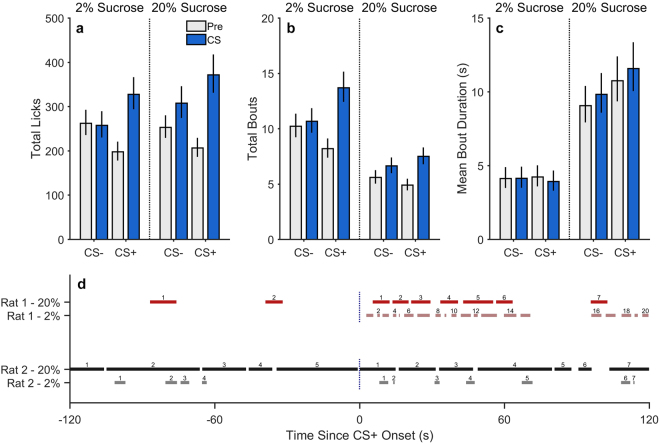
Microstructural components of licking behavior. Collapsed data from all non-drug conditions from Experiments 1–3 assessing the impact of a sucrose-paired cue (CS+) and unpaired cue (CS−) on sucrose consumption. These data represent the mean number of licks (**a**), bouts (**b**), and bout duration (**c**) as a function of CS period (Pre, CS), CS type (+,−), and drug. Error bars represent ±1 standard error of the estimated marginal means from a generalized linear mixed-effects model equivalent in structure to that used for overall lick analysis in Experiments 1–3. (**d**) Representative diagram of changes in bout frequency and duration as a function of CS period (pre-CS+ vs. CS+) and sucrose concentration (2% vs. 20%). Each line represents an individual licking bout. The temporal duration of that bout is represented by the length of that line. The running total of bouts in each period is indicated by the number above the individual bouts.

To ensure sufficient statistical power^39^, we collapsed data across all non-drug test conditions described above (2% and 20% tests for Experiment 1 and Experiment 2, and the vehicle condition for Experiment 3). The combined data are shown in Fig. 2, plotted separately as total licks (a), bout frequency (b), and bout duration (c). Figure 2d shows raster plots of two representative rats’ licking behavior during pre-CS+ and CS+ periods when 2% and 20% sucrose were available at test. In accordance with the motivational interpretation of CPF, these rats tended to engage in more bouts of sucrose licking during the CS+ than during the pre-CS+ period. In contrast, bout durations tended to be longer when the rats were consuming the more palatable 20% sucrose solution than when consuming 2% sucrose, an effect that was apparent during pre-CS+ and CS+ periods. Thus, bout duration was not strongly influenced by the sucrose-paired cue. Indeed, the patterns seen in Fig. 2d were corroborated by generalized linear mixed-effects models of the combined data set (see Fig. 2a–c and Supplementary Table S4). Secondary mixed-effects analyses revealed that the categorical factor of “Experiment” (1, 2, 3) did not significantly moderate the CS Period × CS Type interactions on bout frequency or duration, *p*s ≥ 0.293, permitting us to combine these data for subsequent analyses. Interestingly, the ability of the CS+ to motivate licking behavior was also reflected in a significantly faster latency to initiate licking^40–42^ after CS+ vs. CS− onset [generalized linear mixed-effects model (response distribution = gamma, link function = log); *t*(306) = −2.71, *p* = 0.007], though the raw difference in latencies was relatively modest (CS+: 1.16 seconds ± 0.47; CS−: 2.79 seconds ± 0.79).

#### Mediational analysis of CS period effect

**Figure 3:**
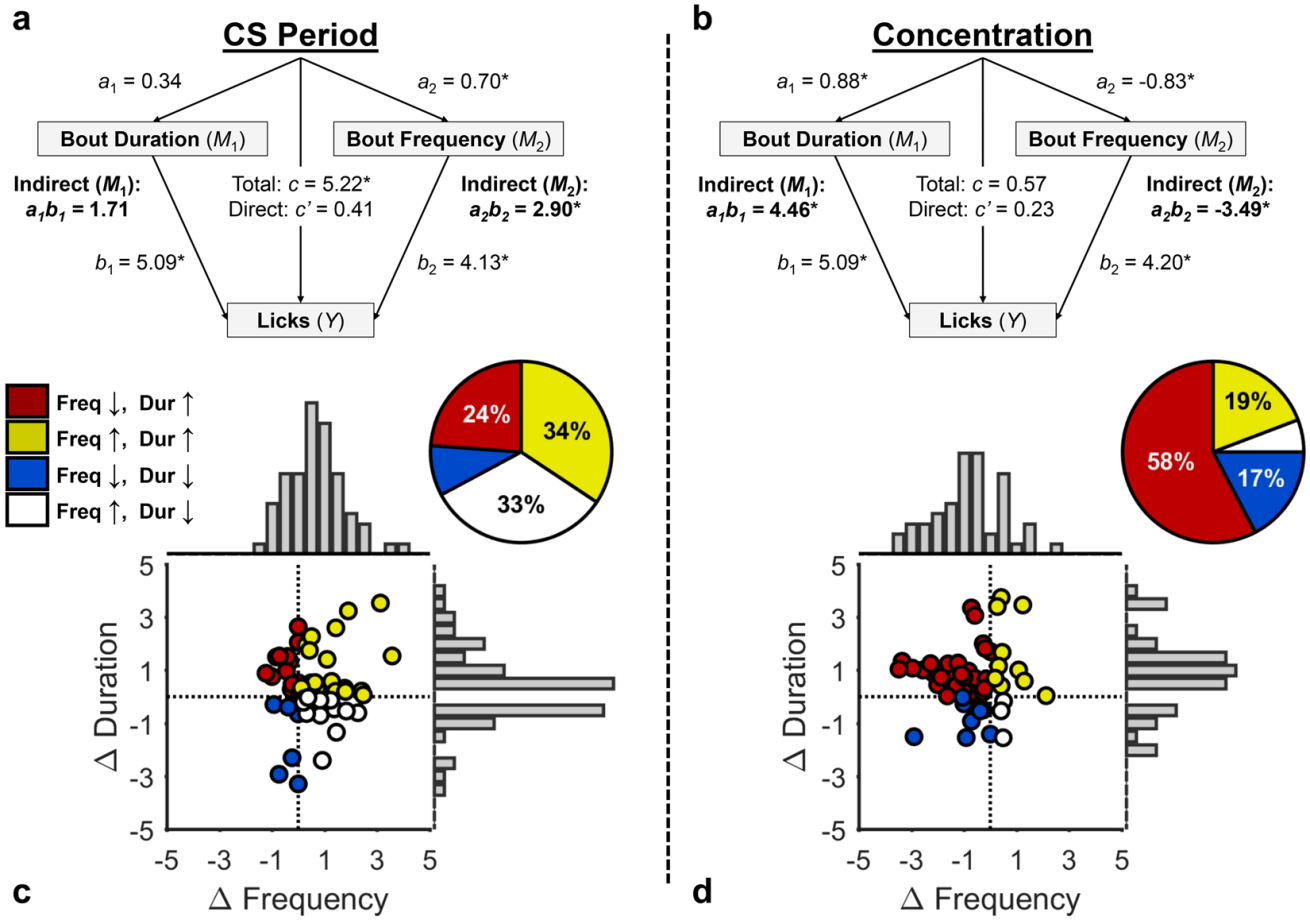
Mediation of CPF by microstructural characteristics of licking behavior. (**a**) CS Period Model describing the effect of CS period on total licks with mediators of bout duration and bout frequency. (**b**) Concentration Model describing the effect of sucrose concentration on total licks with mediators of bout duration and bout frequency. (**c**) The change in bout frequency and bout duration from pre-CS+ to CS+ periods. (**d**) The change in bout frequency and bout duration from 2% to 20% sucrose concentration tests. (**c**,**d**) The frequency histogram above and to the right of each panel refers to the bout frequency and bout duration data, respectively. The pie chart reflects the proportion of data points in each quadrant of the Cartesian plane. Each quadrant of the scatter plot and segment of the pie chart are color coded to reflect whether bout frequency (Freq) and duration (Dur) increased (↑) or decreased (↓) as a function of CS period (**a**,**c**) and concentration (**b**,**d**). There was strong correspondence between the observed and expected number of data points in each quadrant across experiments based on the overall distribution data (CS Period: *r* = 0.91, *p* < 0.001; Concentration: *r* = 0.96, *p* < 0.001).

Given such findings, we performed a statistical mediation analysis^43^ on the combined data (Fig. 2) to determine if CS+ evoked sucrose drinking was preferentially related to changes in bout frequency or duration. Figure 3a shows the multiple mediation model structure for this analysis (CS Period). There was a significant overall effect (Total; *c*) of CS period on licking behavior, *t*(156) = 4.11, *p* < 0.001, *c* = 5.22 [2.71, 7.73], in that there were more licks during the CS+ than the pre-CS+ period. We then tested whether the CS+ similarly influenced licking microstructure, and found a significant cue-induced elevation in bout frequency (*M*_2_), *t*(156) = 3.27, *p* = 0.001, *a*_2_ = 0.70 [0.28, 1.12], but not bout duration (*M*_1_), *t*(141) = 1.89, *p* = 0.061, *a*_1_ = 0.34 [−0.02, 0.69]. Thus, at a group-level, the CS+ effect on bout frequency, but not bout duration, resembled its effect on licking more generally.

If the effect of the CS+ on licking was mediated by its effect on bout frequency, then (1) these measures should be correlated, and (2) the CS+ effect on bout frequency should account for the CS+ effect on the total licks measure. An assessment of the first prediction found that, ignoring CS period, both bout frequency and bout duration were significantly correlated with total licks, *p*s < 0.001, which is unsurprising given that these microstructural measures bear an intrinsic relationship with total licks. Our assessment of the second prediction, however, was more revealing. We constructed a multiple mediation model to examine if these microstructural measures explained CS+ related variance in the total lick measure by including bout frequency and bout duration as fixed effects, along with CS period. In other words, we asked whether controlling for variance in these lick bout measures weakened the CS+ effect, relative to its strength in the simpler (reduced) model described above. Consistent with mediation, we found that this *direct* effect of CS period on licks (*c*’) was not significant, *t*(139) = 0.90, *p* = 0.370, *c*’ = 0.41 [−0.49, 1.30], when controlling for bout frequency and duration. We then estimated the influence of CS+ on licking through each of these potential mediators, and found that there was a significant indirect effect of bout frequency on licks, *a*_2_*b*_2_ = 2.90 [1.18, 4.76], but not of bout duration, *a*_1_*b*_1_ = 1.71 [−0.09, 3.35]. Thus, these data indicate that CS+ induced elevations in licking are primarily driven by increases in bout frequency as opposed to increases in bout duration, consistent with a motivational rather than a hedonic account of CPF.

#### Mediational analysis of sucrose concentration effect

We performed a second mediational analysis on the combined data (Fig. 2) to confirm that sucrose palatability (concentration) was related to a selective increase in bout duration (Fig. 3b, Concentration). The simplified model (no fixed effects for bout frequency or duration) found that the total effect of concentration on total licks was not significant, *t*(156) = 0.42, *p* = 0.678, *c* = 0.57 [−2.13, 3.27], indicating that overall levels of sucrose licking at test did not strongly depend on sucrose concentration. This is to be expected, since the effect of sucrose palatability on licking is most apparent during the initial 2–3 minutes of consumption^44^, well before the first pre-CS period in our test sessions. Nevertheless, sucrose concentration did have a significant effect on bout duration (*M*_1_), *t*(141) = 5.20, *p* < 0.001, *a*_1_ = 0.88 [0.54, 1.21], with 20% sucrose supporting longer bouts of drinking than 2% sucrose. Interestingly, sucrose concentration had a significant suppressive effect on bout frequency (*M*_2_), *t*(156) = −3.84, *p* < 0.001, *a*_2_ = −0.83 [−1.26, −0.40], in that rats tended to engage in fewer bouts when drinking a more palatable solution. Thus, concentration-related increases in bout duration were offset by decreases in bout frequency. Consistent with this, our full mediation model, which included fixed effects for bout duration and frequency, indicated no direct effect of concentration on licks, *t*(139) = 0.45, *p* = 0.650, *c*’ = 0.23 [−0.76, 1.22]. However, there were significant indirect, but opposite, effects of bout frequency, *a*_2_*b*_2_ = −3.49 [−5.50, −1.58], and bout duration, *a*_1_*b*_1_ = 4.46 [2.96, 5.95], on total licking behavior.

#### Individual differences in the effect of CS period and concentration on licking microstructure

The mediation models revealed that bout frequency and duration play distinct roles in mediating the effects of the CS+ and sucrose concentration on licking at a group-level, but do not address how such effects are expressed across individual rats, which may be important to understanding individual vulnerabilities to overeating. Given the results of the mediation analysis, we predicted that individual rats would show a net increase in bout frequency during the CS+ period, relative to baseline, but would not show any consistent or reliable change in bout duration. Furthermore, individual rats were predicted to show longer, but less frequent, bouts of licking when consuming 20% sucrose, relative to the 2% test. Fig. 3c and d show individual differences in the effects of CS period (CS+ - pre-CS+) and sucrose concentration (20%–2%), respectively, on bout frequency and duration (analysis of combined data set in Fig. 2). The CS+ increased bout frequency in 67% of rats (Fig. 3c), with roughly equal numbers of these rats also showing an increase in bout duration (34%) or not (33%). A chi-squared goodness of fit test assuming uniformly distributed data points across the four quadrants revealed significant distributional asymmetry, χ^2^(3) = 10.91, *p* = 0.012. Indeed, the mean of the Δ_Frequency_ distribution was significantly greater than 0, *t*(66) = 4.80, *p* < 0.001, while the mean of Δ_Duration_ distribution did not significantly differ from 0, *t*(66) = 1.80, *p* = 0.076. With regard to the concentration effect (Fig. 3d), the majority of rats (58%) exhibited longer *and* less frequent bouts with 20% versus 2% sucrose, and a chi-squared goodness of fit test confirmed that the data were not uniformly distributed across quadrants, χ^2^(3) = 31.85, *p* < 0.001. Indeed, we found that the mean of the Δ_Frequency_ distribution was significantly less than 0, *t*(51) = −4.22, *p* < 0.001, while the mean of the Δ_Duration_ distribution was significantly greater than 0, *t*(51) = 4.18, *p* < 0.001.

#### Microstructural predictors of sucrose consumption

**Figure 4:**
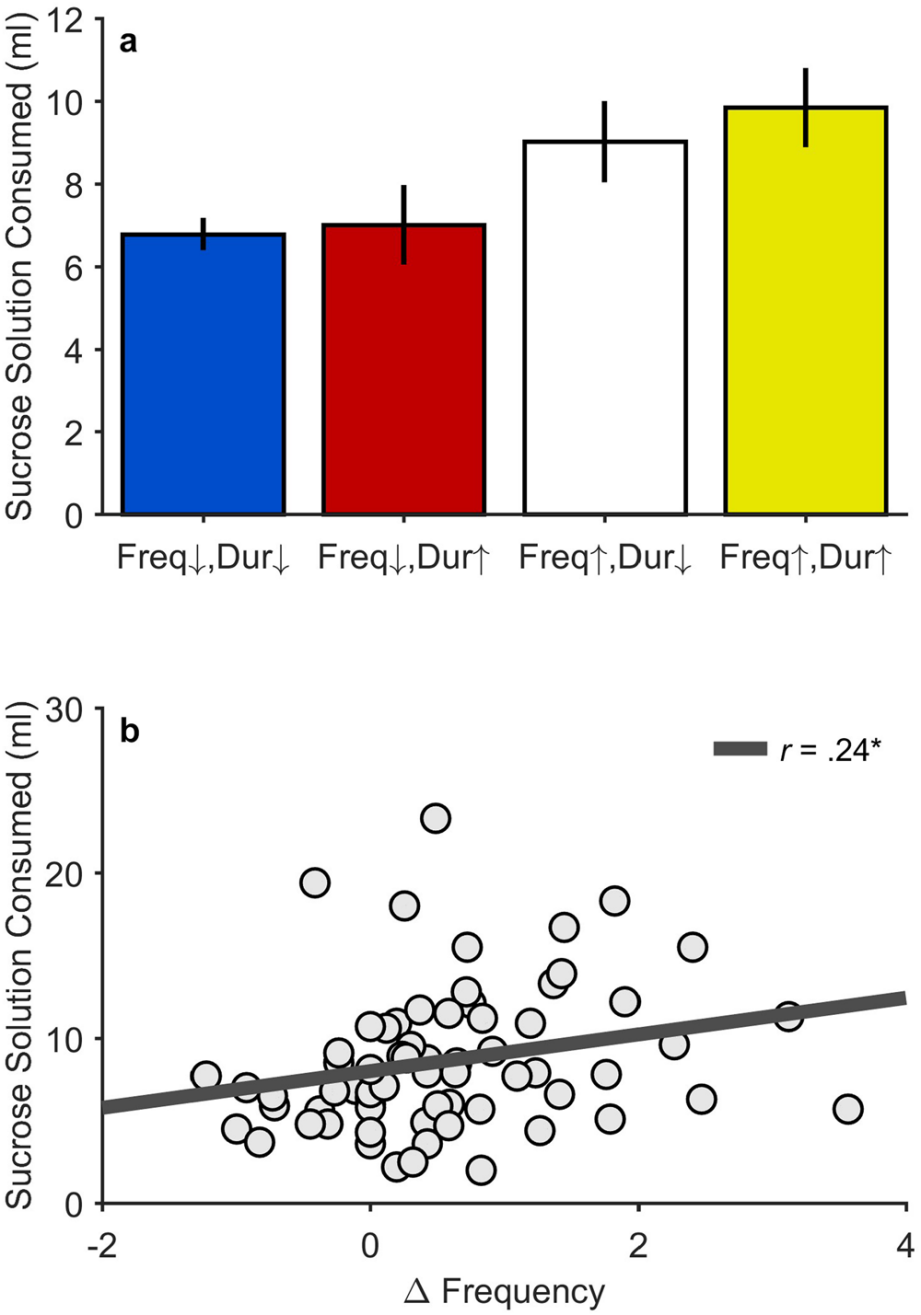
Volume of sucrose solution (ml) consumed as a function of CS+ evoked changes in bout frequency and duration. (**a**) These data represent sucrose consumption as a function of categorical group, determined by CS+ evoked increases (↑) or decreases (↓) in bout frequency (Freq) or bout duration (Dur). Error bars represent ±1 between-subjects standard errors. (**b**) Relationship between CS+ change in bout frequency (Δ_Frequency_) and sucrose solution consumed (ml). The best fitting regression line is shown. *p < 0.05.

The data in Fig. 3c suggest that there was considerable variability in the effect of the CS+ on bout frequency, and that some rats were particularly sensitive to this motivational influence. Although it is possible that these rats were able to control their total sucrose intake by drinking less in the absence of the CS+, further analysis of the combined data set (Fig. 2) confirmed that these CS+ triggered increases in bout frequency were associated with overeating. Specifically, we found that rats who exhibited positive Δ_Frequency_ scores during CS+ trials (subgroups Freq↑, Dur↓ and Freq↑, Dur↑ in Fig. 3C) consumed significantly more sucrose than rats who did not (subgroups Freq↓, Dur↓ and Freq↓, Dur↑), *t*(63) = 2.27, *p* = 0.026 (Fig. 4a). This relationship was maintained when Δ_Frequency_ was treated as a continuous variable, *t*(63) = 2.19, *p* = 0.032 (Fig. 4b), and did not depend on sucrose concentration, Concentration × Δ_Frequency,_ *t*(63) = 0.64, *p* = 0.528.

## Discussion

We found that a cue signaling sucrose availability was able to potentiate sucrose intake in rats regardless of whether that cue also signaled the specific actions needed to obtain sucrose (Experiment 1) or did not (Experiments 2 and 3). The latter finding is of particular interest because it is unlikely to depend on the execution of pre-existing conditioned feeding responses (or stimulus-response habits), and instead suggests that such cues acquire affective and/or motivational properties which allow them to flexibly transfer their control across feeding actions. This tendency for environmental stimuli to promote food consumption even when established feeding routines are not readily available therefore seems to provide a useful and selective animal model of the Pavlovian process that supports cue-elicited food cravings and overeating in humans^1–3^. While there are previous reports that food-paired stimuli can promote feeding in a response-independent manner^14–16^, most CPF experiments keep the food source fixed across training and testing phases, and therefore provide only limited information about the nature of the psychological processes underlying this effect. The current study provides a demonstration of the generalized excitatory influence of food-paired cues on feeding behavior using a procedure modeled after the PIT task, which is widely used to study the generalized motivational influence of food-paired cues on food-seeking behavior. For instance, as in PIT, the current task can be used to assess the tendency for a cue to acquire motivational properties that generalize to a new location. We also borrowed training and testing parameters (e.g., cue duration, inter-trial intervals, and reinforcement schedule) that are commonly used for PIT, facilitating comparisons across studies. This approach may therefore provide greater experimental control for future investigations of potential differences in the psychological and/or biological processes underlying Pavlovian control over instrumental vs. consummatory behavior.

The current study found that activation of D1 dopamine receptors is critical for the expression of this response-independent form of CPF, which helps support an incentive motivational interpretation given the importance of dopamine signaling generally, and D1 receptor activation specifically in the expression of Pavlovian-to-instrumental transfer^18,29–32,45,46^. Given evidence that dopamine is relatively unimportant for processing the hedonic properties of food stimuli^25,28,34^, it seems unlikely that the D1 antagonist had its effect by disrupting the capacity of the CS+ to alter sucrose palatability at test. This motivational interpretation is also supported by our microstructural lick analysis, which found that the cues increased feeding primarily by eliciting more bouts of licking, rather than by extending the duration of those bouts. Instead, bout durations varied with sucrose palatability, as has been well established^23,24,26,27^. Interestingly, our statistical mediation analysis revealed that although rats engaged in longer bouts when licking 20% vs. 2% sucrose, they also showed a compensatory decrease in bout frequency. Therefore, this manipulation of palatability seemed to affect the way rats patterned their sucrose intake without impacting their overall level of feeding. In contrast, no such compensatory effect was evident during trials with the CS+, which would seem to account for the net increase in licking behavior observed on trials with that cue. Furthermore, rats showing increases in bout frequency during CS+ trials also showed heightened levels of total sucrose intake. Such findings suggest that food-paired cues (1) can dysregulate feeding behavior, and (2) are more effective in driving overeating than manipulations of sucrose palatability, at least under the conditions tested here.

The current results also shed light on the role of dopamine in the regulation of feeding behavior in the absence of explicit food-paired cues. Previous studies have shown that systemic administration of the D1 dopamine antagonist SCH23390 suppresses *uncued* sucrose consumption by reducing bout frequency without altering bout duration^25,26^, which is similar to the pattern of licking exhibited by dopamine deficient mice^47^. Though the psychological mechanisms controlling bout frequency in such situations are not clear, it has been suggested that contextual and/or interoceptive cues that have become associated with feeding acquire the ability to surreptitiously motivate new bouts of food seeking and consumption^25,26^. Our results provide some support for the plausibility of this interpretation by demonstrating that new bouts of licking can be elicited by explicit food-paired cues and that this effect also depends on D1 dopamine receptor activation.

As noted elsewhere^5,10^, there has been relatively little previous research on the role of dopamine in CPF. However, one early study found that administration of the nonspecific dopamine receptor antagonist α-flupenthixol attenuated CS+ elicited food seeking but left intact that cue’s ability to increase food consumption^48^, which seems to be at odds with our finding that D1 antagonism disrupts cue-induced sucrose licking. There are numerous procedural differences across the two studies that could explain this apparent discrepancy. For instance, it may be that our selective manipulation of D1 dopamine transmission is more effective in disrupting the influence of CS+ on food intake. Furthermore, in this previous study^48^, food-deprived rats were trained and tested in their home cages using a unique Pavlovian conditioning procedure in which a cue was used to signal feeding sessions that were distributed intermittently throughout the day. Later, that cue was shown to be effective in promoting feeding even when rats were tested in a nondeprived state. The nature and extent of this training and the fact that the required feeding responses were unchanged across training and testing phases suggest that this CPF protocol may have encouraged the use of a habitual (stimulus-response) feeding response during testing. Given that overtraining can render cue-evoked food seeking insensitive to manipulations of dopamine signaling^49^, it may be that this potentially habit-based form of CPF is less dependent on dopamine than the motivational form described here.

Although much remains to be determined about the role of dopamine in CPF, this behavioral phenomenon is known to depend on the ghrelin^50–52^ and melanin-concentrating hormone^53^ neuropeptide systems, which are fundamentally involved in regulating both feeding behavior^10^ and dopamine signaling^54–56^. Interestingly, ghrelin’s appetite-stimulating effects depend on this hormone’s ability to modulate mesolimbic dopamine signaling^57–60^. For instance, the tendency for ghrelin to enhance food seeking and consumption without affecting food palatability (licking bout duration) can be inhibited by co-administering the D1 dopamine receptor antagonist SCH-23390^60^. Based on such findings, one might expect that a similar interaction between ghrelin and dopamine may underlie the motivational influence of food-paired cues over feeding.

While the current findings demonstrate that food-paired cues can stimulate overeating by motivating new bouts of feeding, such cues are also likely to influence feeding through other processes. Implicit in our transfer-of-control approach is the recognition that feeding cues can trigger intake by directly eliciting specific feeding behaviors. Furthermore, although the CS+ did not reliably alter bout durations in the current study, a recent study employing a more conventional CPF protocol with a fixed food source did find evidence that feeding cues can elongate licking bouts^53^. In line with this, there are previous reports that cues associated with palatable food can augment expression of appetitive orofacial reactions to taste stimuli^19–21^, another measure of taste hedonics or “liking”. Thus, it is likely that food cues can prompt feeding through multiple routes, by causing cravings, by triggering specific feeding responses, and/or by making food taste better^10^. These processes may underlie distinct vulnerabilities to cue-potentiated overeating, perhaps explaining individual differences in susceptibility to this effect^1,61,62^. The current findings demonstrate an effective approach for selectively parsing the motivational component of CPF in rats.

## Methods

### Subjects and apparatus

Adult male Long Evans rats (N = 32 total rats; n = 16 for Experiment 1 and n = 16 for Experiments 2 and 3), weighing 370–400 g upon arrival, were pair housed in transparent plastic cages in a temperature- and humidity-controlled vivarium. Rats had *ad libitum* access to water in their home cages throughout the experiment. Rats were placed on a food restriction schedule during certain phases of the experiment, as specified below. Husbandry and experimental procedures were approved by the UC Irvine Institutional Animal Care and Use Committee (IACUC) and were in accordance with the National Research Council Guide for the Care and Use of Laboratory Animals.

Behavioral procedures were conducted in identical chambers (ENV-007, Med Associates, St Albans, VT, USA), housed in sound- and light-attenuated cubicles. Sucrose solution could be delivered via a syringe pump into a recessed plastic cup that was centrally located in one end wall of each chamber, 2.5 cm above the stainless-steel grid floor. A photobeam detector positioned at the entrance of the food receptacle was used to monitor head entries associated with sucrose consumption, as well as conditioned approach responses during Pavlovian conditioning sessions. In certain test sessions (Experiments 2 and 3), sucrose solution could be obtained by licking a gravity-fed metal drinking spout that was positioned ~0.5 cm into a 1.3 cm hole located on the end wall opposite to the food cup. Individual licks from the food cup and metal spout were continuously recorded during test sessions using a contact lickometer device (ENV-250B, Med Associates, St Albans, VT, USA). A white opaque plexiglass panel was positioned in front of the end wall housing the food cup during all sessions when sucrose could be obtained from the metal spout. A houselight (3 W, 24 V) provided illumination and a fan provided ventilation and background noise.

### Pavlovian conditioning

Rats were placed on a food restriction schedule to maintain their body weights at approximately 85% of their free-feeding body weights prior to undergoing 2 d of magazine training, in which they received 60 deliveries of 20% sucrose solution (0.1 ml) in each daily session (1 h). Rats then received 10 d of Pavlovian conditioning. Each daily conditioning session consisted of a series of 6 presentations of a 2-min audio cue (CS+; either 80-dB white noise or 10-Hz clicker), with trials separated by a variable 3-min interval (range 2–4). During each CS+ trial, 0.1 ml aliquots (delivered over 2 sec) of 20% sucrose solution (w/v) were delivered into the food cup according to a 30-sec random time schedule, resulting in an average of four sucrose deliveries per trial. On the last day of conditioning, rats were also given a second session in which the alternative cue (CS−; alternative auditory stimulus) was presented in the same manner as the CS+ but was not paired with sucrose solution. Anticipatory behavior was measured by comparing the rate of cup approaches (photobeam breaks) during the period between the CS onset and the first sucrose delivery (to avoid detection of unconditioned feeding behavior), which was contrasted with the rate of cup approaches during the inter-trial interval. All rats were then given five days of *ad libitum* access to their maintenance diet after the last session of Pavlovian conditioning before undergoing additional testing.

### Cue-potentiated feeding test

#### Experiment 1

This experiment assessed the impact of the CS+ on the consumption of sucrose solution from the same food cup used during training, such that the conditioned response to that cue (i.e., cup approach) was compatible with the behavior needed to obtain sucrose at test. After regaining weight lost during the Pavlovian conditioning, rats received a pair of CPF tests, which were separated by 48 h, during which rats remained undisturbed in their homecages. During each CPF session (86 min in total duration), 2% or 20% sucrose solution was continuously made available in the food cup by refilling that cup with 0.1 ml of sucrose whenever the rat crossed the photobeam (cup approaches). However, to prevent overfilling the cup, the sucrose delivery was only administered if at least 4 s had elapsed since the last sucrose delivery and if the rat had performed at least five licks during the intervening period. Over the course of this session, each of the 2-min auditory stimuli was noncontingently presented 4 times in a pseudorandom order (ABBABAAB), separated by a fixed 8-min interval. The first trial began 8 min after the start of the session to allow for the induction of satiety prior to assessing the behavioral influence of the cues. Trial order was counterbalanced with Pavlovian training conditions, such that the first CS presented was the CS+ for half of subjects and the CS− for the remaining half of subjects. The order of sucrose concentration testing was also counterbalanced, with half of each condition receiving the 2% test first and the 20% test second, and half receiving the opposite arrangement (i.e., all animals received both concentrations in separate tests).

#### Experiment 2

In this experiment, we investigated the effect of the CS+ on consumption of sucrose solution from a different source than the cup used during Pavlovian conditioning, such that the conditioned response to that cue was incompatible with behavior needed to consume sucrose at test. The first test we conducted included only the 2% sucrose condition. After allowing rats to regain weight lost during Pavlovian conditioning, they were given two daily sessions (86 min duration) in which they had unrestricted access to 2% sucrose solution from a metal spout (gravity-fed via bottle) positioned within a small hole in the end-wall opposite the food cup. A white Plexiglas panel was positioned in front of the wall housing the food cup during sessions with spout access (including subsequent CPF tests) to discourage animals from searching for sucrose at this location. These sessions were designed to give rats experience drinking sucrose from a new source in the absence of the auditory cues. On the following day, rats received a single CPF test session as described in Experiment 1, except that 2% sucrose was continuously available at the metal spout, rather than at the cup.

Because there was little evidence of CPF in this first test, presumably due to response competition between CS+ evoked food cup and spout approach behavior, we gave rats additional spout training (in the absence of the CS+) to strengthen sucrose seeking at the spout and discourage food cup approach when the spout was available (because it was covered with a panel). Rats were therefore placed back on a food restriction schedule (same as during Pavlovian conditioning phase) before being given 5 d of additional spout training sessions, with each of these sessions consisting of 10 min of access to 20% sucrose solution. Rats were then given 4 d of *ad libitum* access to home chow to allow them to regain weight lost during this phase. Next, rats were acutely food deprived (20 h) prior to receiving Pavlovian retraining sessions with the CS+ and CS−, as during the last day of initial training (i.e., with 20% sucrose delivered into food cup during CS+ trials). Note that the spout was removed from the chamber during these and all subsequent Pavlovian retraining sessions. Rats were then given ~20 h of *ad libitum* access to home chow prior to undergoing two CPF tests using the metal spout, which were identical to the first test, except that rats were given access to 2% or 20% in two separate tests (as in Experiment 1).

#### Experiment 3

After finding more substantial evidence of CPF during the last round of testing with the spout, rats from Experiment 2 were given additional testing to assess the dependence of this effect on dopamine signaling at D1 dopamine receptors. Rats were first given a 10-min session of spout retraining in which they were given access to 20% sucrose solution. Because rats rapidly returned to normal bodyweight when returned to *ad libitum* home chow following acute 20-h food deprivation, we used this procedure to ensure that rats were hungry during this spout retraining session and during subsequent Pavlovian retraining (CS+ and CS− sessions, as before), which was conducted on the day before each of the two final CPF tests. Rats were given at least 20 h of *ad libitum* home chow access before each test session. During this final round of CPF testing, rats had continuous access to 20% sucrose from the spout during both test sessions. Fifteen min before each test, rats were given an i.p. injection (1 ml/kg) of either sterile saline or SCH-23390 (selective D1 dopamine receptor antagonist) using a dose (0.04 mg/kg) known to be sufficient to suppress sucrose consumption^25,34,63^. Rats were tested in both drug conditions, counterbalancing for test order.

### Data analysis

The primary dependent measure was individual licks, which were recorded with a 10-ms resolution using a contact lickometer during all CPF sessions. Very rarely, we detected artifacts in our lickometer measurements that were caused by sustained contact between the rat (paw or mouth) and sucrose (or metal spout). These artifacts took the form of high-frequency lickometer responses (>20 Hz). Given that rats exhibit a maximal licking rate of <10 Hz^64^, we excluded all potential lick responses occurring within 0.05 sec of the last (non-artifact) lick, corresponding to a 20-Hz cutoff frequency. Sessions in which at least 20% of lick responses were excluded given this criterion were altogether removed from analysis (1 session from 1 rat in Experiment 1).

#### Licking behavior

For each session, we determined the total number of licks across period types (Pre-CS+, CS+, Pre-CS−, CS−). Because our primary dependent measure (total licks) is a count variable, these data were analyzed using generalized linear mixed-effects models with a Poisson response distribution and a log link function ^65-68^^.^ This statistical approach allows for parameter estimation as a function of condition (fixed effects) and the individual (random effects). In Experiments 1 and 2, the fixed-effects structure included an overall intercept, the three-way interaction between CS Period (Pre, CS) × CS Type (CS−,CS+) × Concentration (2%, 20%), and all lower-order main effects and interactions. For Experiment 3, Drug (Vehicle, SCH) was substituted in for Concentration to accommodate the change in experimental design. These variables were all within-subjects variables, treated as categorical predictors, and effects-coded. Random-effects model selection involved determining the model that minimized the Akaike information criterion ^69^, while also ensuring that the number of data points per parameter did not fall below 10 ^70,71^. Using these criteria, the best random-effects structure across experiments included by-subjects uncorrelated intercepts adjusted for CS Period, CS Type, and Concentration (or Drug)^72^. All statistical analyses were conducted in MATLAB (The Math Works; Natick, MA). The alpha level for all tests was 0.05. As all predictors were categorical, effect size was represented by the unstandardized regression coefficient ^73^, reported as *b* in-text and in model output tables. Post hoc analyses of interactions were conducted using post hoc *F*-tests of the simple effects within the omnibus analysis using the *coefTest* function in MATLAB.

#### Microstructural analysis of licking behavior

Individual licks were categorized as either beginning or continuing a licking bout. A bout was demarcated as multiple consecutive licks in which the interlick intervals (ILI) did not exceed 1 s^74^. When at least 1 s had passed from the last lick, the next lick was designated as the beginning of a new bout. Bout frequency and duration were calculated by first partitioning the sessions into pre-CS and CS periods, as done for total licks in the analyses above. In those periods, every lick that was preceded by a period of at least 1 s was designated as a bout. The duration of each bout was calculated as the time interval between the first and last lick in that bout. Individual licks occurring in isolation were not counted as being part of a bout. To maximize sample size for the subsequent mediational analyses^39^, the bout frequency and bout duration data were collapsed across experiments to evaluate general effects of CS period, CS type, and concentration on these microstructural measures. Data from the SCH-23390 condition in Experiment 3 were not included in these analyses.

These data were analyzed via generalized linear mixed effects models incorporating a fixed-effects structure of CS Period × CS Type × Concentration (and all lower order interactions and main effects) and a random-effects structure of by-subjects uncorrelated intercepts adjusted for CS Period, CS Type, and Concentration. As in the analysis of total licking behavior, one session for one rat from Experiment 1 was removed from the analysis. The bout frequency analysis employed a Poisson response distribution with a log link function due to the count-type nature of frequency data. The bout duration analysis employed a gamma response distribution with a log link function as bout duration is a continuous measure bounded between 0 and + ∞. For comparison, this same analysis was run on total licks collapsed across experiments, in which the analysis assumed a Poisson response distribution with a log link function as in the individual experiment total lick analyses. To ensure that the critical CS Period × CS Type interaction did not depend on which experiment each rat was in, a second series of models were run on the bout frequency and bout duration, identical to the analysis just described but with an additional fixed effect predictor of Experiment × CS Period × CS Type. Experiment was a categorical factor. Lastly, as a confirmatory measure of motivated licking^40–42^, we analyzed the latency to the first lick following CS onset using a generalized linear mixed-effects model with a gamma response distribution and a log link function (*n* = 310). This model included a fixed-effects structure of CS Type × Concentration (and all lower order interactions and main effects) and a random-effects structure of by-subjects intercepts adjusted for CS Type, Concentration, and CS Type × Concentration.

#### Mediational analysis of bout frequency and bout duration

Two multiple mediation models^43,75,76^ were conducted to determine whether effects (or lack thereof) of CS period (Pre, CS) and concentration (2%, 20%) on CPF were significantly mediated by bout frequency and/or bout duration. In the CS Period Model, the variable *X* was CS period (Pre, CS), the outcome variable *Y* was the total number of licks in that period, and the mediators were bout frequency (*M*_1_) and bout duration (*M*_2_). In the Concentration Model, the variable *X* was sucrose concentration. Because cue-evoked licking was primarily evident for CS+ trials (see *Results*), only CS+ trials were analyzed. For each rat and for each test session, the average number of licks and bouts and the average duration of each bout was determined for pre-CS+ and CS+ periods. These analyses included all rats from Experiments 1 and 2 (16 rats per experiment × 2 experiments × 2 concentrations × 2 CS periods = 128 data points) and the vehicle condition data from Experiment 3 (16 rats × 2 CS periods = 32 data points). As in the analysis of total licking behavior, one session for one rat from Experiment 1 was removed from the analysis, leaving a total of 158 data points. Rarely, rats did not lick during the pre-CS+ or CS+ periods during a session (9/158; 9.5%). In these instances, the average number of licks and bouts were coded as “0” and the value for average bout duration was left as an empty cell. When the same models were run assuming listwise deletion (i.e., removing rows in which bout duration was an empty cell), similar patterns held. Because these analyses involve general linear models (i.e., simple or multiple linear regression), the bout frequency and total lick data were square-root transformed and the bout duration data were log-transformed to correct for positive skewness. Significance of the indirect effect was determined by 95% percentile bootstrapping with 10,000 iterations. Regression coefficients are reported in correspondence with traditional mediational analysis reports (e.g., *c*’ = direct effect of *X* on *Y*)^43,75^.

#### Individual differences in cue-evoked changes in bout frequency and duration

The aforementioned analyses allowed us to assess the effect of the CS+ on licking microstructure at the group level. We also characterized individual differences in the expression of this effect. For each rat, two difference scores were computed for the bout frequency and bout duration measures. As a parallel to the CS Period Model, bout frequency during the pre-CS+ period was subtracted from the bout frequency value during the CS+ period (i.e., CS+ - pre-CS+); for the Concentration Model, bout frequency during the 2% sucrose test was subtracted from the corresponding value during the 20% test (i.e., 20%–2%). These computations produced measures describing the change in bout frequency (Δ_Frequency_). These same calculations were done for bout duration (i.e., Δ_Duration_). Thus, for each pair of Pre-CS+/CS+ and 2%/20% data points, changes in bout frequency and bout duration were determined. The means of these distributions were compared to 0 via a one-sample *t*-test (α = 0.05) to evaluate distributional shifts away from no general change. Each of these data points were categorized by increases and/or decreases in bout frequency and duration and represented by a bivariate scatter plot (e.g., increase in bout frequency/decease in bout duration upon CS+ onset), allowing for determination of the proportion of data points in each 2 × 2 quadrant (bout frequency/duration × increase/decrease). Data points in which the difference score was equal to zero was categorized as a decrease (i.e., not an increase). Chi-squared (χ^2^) goodness of fit tests for both the CS period and concentration data determined whether the distributions of these data points were different from uniformly-distributed data across these four categories (α = 0.05). To determine if there was an approximately equal distribution of these data points across the four quadrants for each experiment, simple correlational analyses for the CS Period and Concentration data were performed to evaluate the relationship between the number of data points in each quadrant in each experiment and the corresponding expected number of data points, as estimated by the overall proportions in each quadrant.

#### Microstructural predictors of sucrose consumption

A final series of generalized linear mixed effects analyses were conducted to determine if the total volume of sucrose solution consumed across entire test sessions was predicted by that rat’s change in bout frequency and duration from the pre-CS+ to CS+ periods. Analyses included data from all non-drug conditions, (i.e., 2% and 20% sucrose tests for Experiments 1 and 2, and the vehicle condition from Experiment 3). The analyses assumed a gamma response distribution with a log link function. The first analysis regressed total sucrose solution consumed (mL) on the main effects of and interactions between the 2 × 2 categorical groupings of increases/decreases in bout frequency/duration as described above. The second analysis regressed total sucrose consumption on the main effects of and interaction between the continuous value of Δ_Frequency_ and sucrose concentration.

### Data availability

The datasets analyzed during the current experiments are available from the corresponding author on reasonable request.

## Electronic supplementary material

Statistical Output Tables
