# Supplementary material for: Contributions of Pavlovian incentive motivation to cue-potentiated feeding
Source: Sci Rep. 2018 Feb 9;8:2766. doi: 10.1038/s41598-018-21046-0 (PMC5807356; doi:10.1038/s41598-018-21046-0)
Supplement: Supplementary file 1 — Statistical Output Tables [file 41598_2018_21046_MOESM1_ESM.pdf]

Contributions of Pavlovian incentive motivation to cue-potentiated feeding

Andrew T. Marshall, Briac Halbout, Angela T. Liu, and Sean B. Ostlund

Table S1. Generalized linear mixed-effects model output for the analysis of cue-potentiated feeding in Experiment 1.

	t(116)	*P*	*b*	95% CI
Intercept	23.70	< .001	5.30	[4.85, 5.74]
Concentration	1.39	.168	0.26	[-0.11, 0.64]
CS Period	3.06	.003	0.15	[0.05, 0.24]
CS Type	-0.38	.706	-0.03	[-0.19, 0.13]
Concentration * CS Period	-2.19	.031	-0.01	[-0.02, -0.001]
Concentration * CS Type	-21.90	< .001	-0.13	[-0.15, -0.12]
CS Period * CS Type	12.70	< .001	0.07	[0.06, 0.08]
Concentration * CS Period * CS Type	-7.18	< .001	-0.04	[-0.05, -0.03]

Note: Categorical variables were effect coded with 2%/20% (Concentration) as -1/+1, Pre-CS/CS (CS Period) as -1/+1, and Unpaired/Paired (CS Type) as -1/+1. Analysis included 124 data points. CS = conditioned stimulus.

Table S2. Generalized linear mixed-effects model output for the analysis of cue-potentiated feeding in Experiment 2.

	t(120)	*P*	*b*	95% CI
Intercept	23.54	< .001	4.98	[4.56, 5.39]
Concentration	-1.14	.255	-0.22	[-0.61, 0.16]
CS Period	2.66	.009	0.27	[0.07, 0.46]
CS Type	-0.77	.440	-0.06	[-0.22, 0.10]
Concentration * CS Period	9.13	< .001	0.06	[0.05, 0.08]
Concentration * CS Type	9.84	< .001	0.07	[0.05, 0.08]
CS Period * CS Type	15.16	< .001	0.10	[0.09, 0.11]
Concentration * CS Period * CS Type	-1.00	.319	-0.01	[-0.02, 0.01]

Note: Categorical variables were effect coded with 2%/20% (Concentration) as -1/+1, Pre-CS/CS (CS Period) as -1/+1, and Unpaired/Paired (CS Type) as -1/+1. Analysis included 128 data points. CS = conditioned stimulus.

Table S3. Generalized linear mixed-effects model output for the analysis of cue-potentiated feeding in Experiment 3.

	t(120)	*P*	*b*	95% CI
Intercept	15.17	< .001	4.86	[4.23, 5.50]
Drug	-2.15	.034	-0.42	[-0.81, -0.03]
CS Period	1.94	.055	0.26	[-0.01, 0.53]
CS Type	0.94	.351	0.12	[-0.14, 0.39]
Drug * CS Period	-19.29	< .001	-0.12	[-0.14, -0.11]
Drug * CS Type	-28.69	< .001	-0.19	[-0.21, -0.18]
CS Period * CS Type	-1.47	.145	-0.01	[-0.02, 0.003]
Drug * CS Period * CS Type	-20.91	< .001	-0.13	[-0.14, -0.11]

Note: Categorical variables were effect coded with Vehicle/SCH23390 (Drug) as -1/+1, Pre-CS/CS (CS Period) as -1/+1, and Unpaired/Paired (CS Type) as -1/+1. Analysis included 128 data points. CS = conditioned stimulus.

Table S4. Generalized linear mixed-effects model output for the combined overall and microstructural analyses of cue-potentiated feeding in Experiments 1 and 2 and the vehicle condition in Experiment 3.

	t(df)	*P*	*b*	95% CI
**Total Licks (df = 308)**				
Intercept	64.14	< .001	5.47	[5.30, 5.64]
CS Period	4.18	< .001	0.20	[0.11, 0.30]
CS Type	-0.21	.833	-0.01	[-0.09, 0.07]
Concentration	0.54	.592	0.03	[-0.09, 0.15]
CS Period * CS Type	29.01	< .001	0.11	[0.10, 0.11]
CS Period * Concentration	15.81	< .001	0.06	[0.05, 0.07]
CS Type * Concentration	3.69	< .001	0.01	[0.01, 0.02]
CS Period * CS Type * Concentration	-4.55	< .001	-0.02	[-0.02, -0.01]
**Total Bouts (df = 308)**				
Intercept	23.84	< .001	2.00	[1.84, 2.17]
CS Period	7.09	< .001	0.14	[0.10, 0.18]
CS Type	-0.30	.761	-0.01	[-0.09, 0.06]
Concentration	-5.72	< .001	-0.27	[-0.36, -0.18]
CS Period * CS Type	4.46	< .001	0.09	[0.05, 0.13]
CS Period * Concentration	0.21	.830	0.004	[-0.04, 0.04]
CS Type * Concentration	-0.50	.617	-0.01	[-0.05, 0.03]
CS Period * CS Type * Concentration	-1.35	.177	-0.03	[-0.07, 0.01]
**Bout Duration (df = 277)**				
Intercept	24.72	< .001	1.83	[1.68, 1.97]
CS Period	0.65	.517	0.03	[-0.06, 0.13]
CS Type	0.94	.347	0.05	[-0.05, 0.14]
Concentration	5.48	< .001	0.42	[0.27, 0.58]
CS Period * CS Type	0.06	.953	0.003	[-0.09, 0.10]
CS Period * Concentration	0.59	.557	0.03	[-0.07, 0.13]
CS Type * Concentration	1.24	.214	0.06	[-0.04, 0.16]
CS Period * CS Type * Concentration	0.14	.890	0.01	[-0.09, 0.10]

Note: Categorical variables were effect coded with 2%/20% (Concentration) as -1/+1, Pre-CS/CS (CS Period) as -1/+1, and Unpaired/Paired (CS Type) as -1/+1. CS = conditioned stimulus.
